# Antibiotic prophylaxis and incidence of infection following elbow arthroplasty: a nationwide study

**DOI:** 10.2340/17453674.2025.43288

**Published:** 2025-03-21

**Authors:** Johan E WÄNSTRÖM, Anne DETTMER, Hanna C BJÖRNSSON HALLGREN, Björn SALOMONSSON, Oskar LJUNGQUIST, Lars E ADOLFSSON

**Affiliations:** 1 Department of Orthopaedic Surgery, Helsingborg Hospital, Helsingborg; 2 Division of Orthopaedic Surgery Department of Biomedical and Clinical Sciences Linköping University, Linköping; 3 Department of Orthopaedic Surgery, Ryhov Hospital, Jönköping; 4 Department of Clinical Sciences, Karolinska Institutet Danderyd Hospital, Stockholm; 5 Department of Clinical Sciences, Lund University Helsingborg Hospital, Helsingborg; 6 Department of Orthopaedics, Faculty of Medicine and Health, Örebro University, Sweden

## Abstract

**Background and purpose:**

Periprosthetic joint infection (PJI) after elbow arthroplasty is a serious complication. Evidence of the best antibiotic prophylaxis for elbow arthroplasty is lacking. We aimed to investigate the regimens presently used in Sweden, incidence of PJI, and the bacteria most frequently found in elbow PJI.

**Methods:**

A questionnaire was sent out to all Swedish units performing elbow arthroplasty in 2019 asking about antibiotic prophylaxis routines. The Swedish Elbow Arthroplasty Register (SEAR) and national inpatient and outpatient registers (NPR) from the National Board of Health and Welfare were searched for procedures related to all primary total- or hemi-elbow arthroplasties performed during 2019–2021. Results of microbiological analyses of the suspected PJI cases were collected from the respective laboratory.

**Results:**

Most centers used only cloxacillin (44%) or cloxacillin together with benzylpenicillin (44%), as prophylaxis. 250 primary procedures were performed between 2019 and 2021, and the most used antibiotic prophylaxes were cloxacillin (61%) and cloxacillin with benzylpenicillin (23%). In the NPR, 20 patients (8%) with a diagnosis that could indicate PJI were found and 9 (3.6%) had a confirmed PJI. The most common bacteria were *Staphylococcus epidermidis, Cutibacterium acnes,* and *Staphylococcus aureus.*

**Conclusion:**

Most centers used cloxacillin antibiotic prophylaxis for elbow arthroplasty. The incidence of PJI was 3.6%. The most frequent diagnosed pathogen was *Staphylococcus epidermidis.*

Elbow arthroplasty is a relatively rare procedure with an incidence rate for total elbow arthroplasty of 1.4 in 100,000 compared with 70–99 in 100,000 for total hip replacement internationally [1,2]. There is a large geographical difference in incidence of elbow arthroplasty and in Sweden less than 100 arthroplasties are performed per year in a population of 10 million [2,3].

Periprosthetic joint infection (PJI) in elbow arthroplasty is a very serious complication that has been reported to occur in up to 12%, which entails significant suffering for the affected patients and substantial strain and financial cost for healthcare [4-6]. The most common causative pathogens are Staphylococcus aureus and Staphylococcus epidermidis [7]. A study by Watts et al., performed in the UK, showed an average of 3.3% PJI in elbow arthroplasty but geographical variation may exist [7]. Described risk factors for infection include young age, rheumatoid arthritis, obesity, previous surgery or infection, and postoperative wound complications [7].

There is a lack of evidence as to the best antibiotic prophylaxis in elbow arthroplasty and no studies that can support a specific prophylaxis for elbow arthroplasty [8]. In Sweden, there is long experience of using cloxacillin as antibiotic prophylaxis in orthopedic surgery, whereas cefazolin, a first-generation cephalosporin with limited gram-negative effect, is generally advocated in international guidelines for joint replacements [8-10].

In the absence of high-quality evidence, orthopedic surgeons follow national recommendations or consensus reports [11]. It is unknown, however, whether it is of advantage to use combination prophylaxis and the best dosage and interval.

We aimed to investigate the different antibiotic regimens recently used in Sweden, incidence of PJI and which pathogen was most frequently found in PJI following primary elbow arthroplasty.

## Methods

### Study design

We performed a nationwide observational study including all centers that perform elbow surgery in Sweden. Register data from the Swedish Elbow Arthroplasty Register (SEAR) and the national inpatient and outpatient registers (NPR), from the National Board of Health and Welfare, were used.

A questionnaire was sent out to all the units where elbow arthroplasty was performed with questions concerning current antibiotic prophylaxis routines. In detail, the questions were which antibiotic prophylaxis was used, how many doses were administered, and for how many days. In addition, the bases for these routines were asked for (giving the alternatives published literature, local recommendations, or advice from infectious disease specialists or own preferences).

The study is reported according to STROBE guidelines.

### Eligibility criteria

Data on all individuals with a primary elbow arthroplasty in 2019–2021 was extracted from the SEAR. The SEAR has a total coverage rate that is calculated by comparing it with the National Board of Health and Welfare’s patient register and is over 90% for total elbow replacement [3]. The NPR register lists all ICD-10 diagnosis codes and the NOMESCO Classification of Surgical Procedures (NCSP) codes, making the extracted data complete. SEAR data was linked to the NPR to identify patients with a reported diagnosis that could indicate infection from 2019 until data extraction in December 2023 according to the ICD-10 and NCSP codes. Only patients operated on between 2019 and 2021 were considered and those found in the NPR register with codes indicative of an elbow infection during the observation time of 2–5 years up to December 2023 were included in the analysis.

### Outcomes

The rate of PJIs in primary elbow arthroplasties registered in 2019–2021 was calculated by dividing all registered individuals diagnosed with a code indicative of an infection (Tables S1 and S2, see Supplementary data) with all registered elbow arthroplasties during the study period. The diagnosis codes sent to the NPR specifically included postoperative infection or other infections, as well as other adverse events regarding the surgical wound, ruptures of tendons, and/or abscesses. The NCSP codes requested from the NPR included surgical exploration, biopsies, operation on bones, incision and debridement of infections, suture of the soft tissue, mobilization, extraction and/or implantation of implants, operation because of bleeding, and other reoperations on soft tissue, bones, and/or implants.

Codes of interest (Tables S1 and S2, see Supplementary data) were sent in advance in connection with the application to the NPR for permission to obtain data. All patients were pseudo-anonymized to a 9-digit number by the registry and all numbers with matching ICD-10 codes that could indicate infection in the elbow were sent back to the NPR to obtain the personal identification numbers for these patients.

The results from the analyses at the respective microbiological laboratory were requested when the personal identification numbers were received.

We defined a periprosthetic joint infection as confirmed if 2 positive cultures with the same microorganism were found [12].

**Table 1:** Number of elbow arthroplasties in the different hospitals in Sweden 2019–2021 according to the Swedish Elbow Arthroplasty Register

Hospital	n (%)
Sahlgrenska University Hospital	59 (24)
Linköping University Hospital	49 (20)
Södersjukhuset	27 (11)
Skåne University Hospital	25 (10)
Academic Hospital Uppsala	22 (8.8)
Varberg	16 (6.4)
S:t Göran	10 (4.0)
Danderyd	9 (3.6)
Gävle	9 (3.6)
Sunderbyn	8 (3.2)
Helsingborg	5 (2.0)
Karolinska	4 (1.6)
Västerås	2 (0.8)
Umeå	2 (0.8)
Huddinge	1 (0.4)
Karlstad	1 (0.4)
Östersund	1 (0.4)
Total	250

### Statistics

All data with patient information to and from the NPR was managed blinded and locked with a password. Demographic data and frequencies were analyzed in SPSS version 26 (IBM Corp, Armonk, NY, USA). Categorical variables were presented as number (n) and percent (%).

### Ethics, funding, use of AI, and disclosures

The study was approved by the Swedish Ethical Review Authority (Dnr 2021-01328 and Dnr 2022-02491-02). JEW was funded by the Stig and Ragna Gorthons’ Fund, Helsingborg, Sweden. HCBH was partially funded by the ALF grant of Sweden (project number 30522028). LEA was partially funded by Linköping University. AI tools were not used in preparation of this manuscript. The authors, their immediate families, and any research foundations with which they are affiliated have not received any financial payments or other benefits from any commercial entity related to the subject of this article. Complete disclosure of interest forms according to ICMJE are available on the article page, doi: 10.2340/17453674.2025.43288

## Results

### Antibiotic regimens

**Figure 1:**
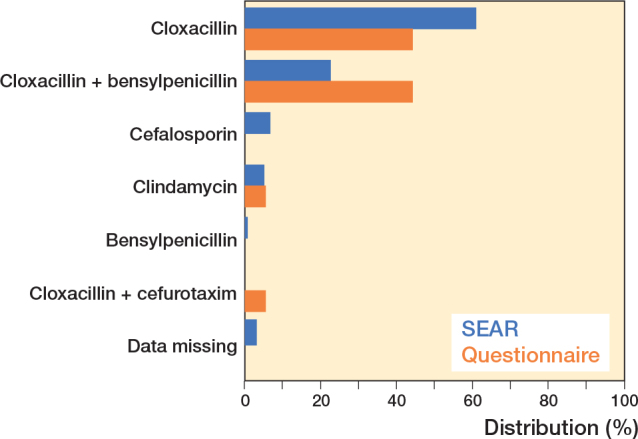
Choice of antibiotic prophylaxis during elbow surgery according to the SEAR and the questionnaire. The data from the Swedish Elbow Arthroplasty Register (SEAR) are from 250 patients and the data from the questionnaire are from 18 hospitals.

The questionnaire was sent to 18 hospitals asking about antibiotic prophylaxis for elbow replacements in 2019, and all 18 responded. 8 hospitals used only cloxacillin, 8 hospitals used cloxacillin combined with benzylpenicillin, 1 hospital used only clindamycin, and 1 used cloxacillin in combination with cefuroxime (Figure 1).

The most common reasons for the antibiotic prophylaxis were recommendation by the local department for infectious diseases (n = 17), recommendation by local colleagues (n = 10), and interpretation of the literature (n = 10) (Table 2). Several reasons were often given by each responder, resulting in more than 18 alternatives.

**Table 2:** Reasons for administered antibiotic prophylaxis, according to the survey

Reason for choice of prophylaxis protocol	n
Recommendation of the local Infection Department	17
Recommendation by colleagues locally	10
Interpretation of the literature	10
Own preferences	9
Consensus at national shoulder and elbow meeting (SSAS, Örebro 2014)	3
Recommendation National Care Programme	1

### Population

According to the SEAR, 250 patients had received a primary elbow prosthesis between 2019 and 2021. The most common reasons for surgery were fresh fracture (67%), rheumatoid arthritis (13%), fracture malunion (8.0%), and primary or secondary osteoarthritis (7.6%). Mean age at the time of operation was 65.5 years and 81% were women. The antibiotics used for prophylaxis according to the SEAR were cloxacillin (61%), cloxacillin and benzylpenicillin (23%), cephalosporin (6.8%), clindamycin (5.2%), and benzylpenicillin (0.8%) (Figure 1). Antibiotics were mostly administered in 3 doses (n = 160; 91%) for 1 day (n = 166; 94%).

**Figure 2:**
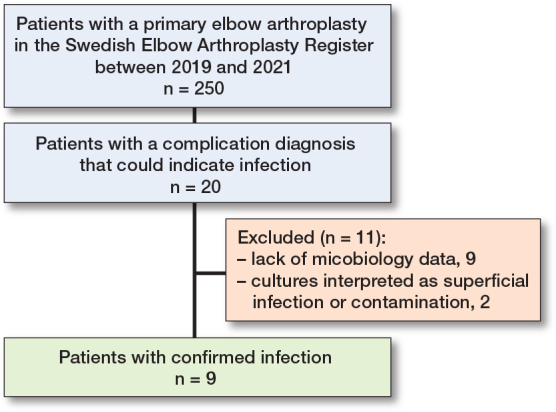
Flowcharts for exclusion of patients with diagnosis of infection.

During the study period, 250 elbow arthroplasties were identified and 20 (8%) of these had a ICD-10 diagnosis or an NCSP code that could indicate an infection. In 9 (3.6 %) of the 20 patients tissue biopsies with microbial cultures demonstrated bacterial growth and were regarded as positively diagnosed infections. In 2 patients, superficial pin cultures showing bacterial growth had been taken and were considered as either superficial infections or contamination and were not included as a PJI (Table 3). For 9 of the 20 patients no data was found in the microbiological laboratories (Figure 2).

**Table 3:** Bacteria identified in the positive cultures

Patient	Bacteria
1	*Staphylococcus* species and *Staphylococcus epidermidis*
2	*Cutibacterium acnes*
3	*Bacillus cereus* and *Staphylococcus epidermidis*
4 **^a^**	Growth of skin flora
5	*Staphylococcus aureus*
6	*Staphylococcus epidermidis*
7	*Staphylococcus epidermidis*
8	Coagulase-negative staphylococci, *Corynebacterium* species and *Cutibacterium acnes*
9	*Staphylococcus aureus*
10	*Cutibacterium acnes*
11 **^a^**	Negative culture

Bacteria growing in cultures from patients with infection.

a Patients 4 and 11 were considered to be contaminations or superficial infection.

The indications for patients with confirmed infection varied; 5 had an acute fracture, 1 rheumatoid arthritis, 1 malunion, 1 secondary arthritis, and in 1 no specific diagnosis was reported to the SEAR. 4 of the patients had had previous fracture surgery. A hemi-prosthesis was used in 2 patients with acute fracture and in 1 patient with malunion; 6 underwent a total arthroplasty.

11 patients with positive cultures were identified in the respective microbiological laboratories. Two of these (patients 4 and 11 in Table 3) had only 1 superficial stick culture each and were therefore not considered as a PJI. 9 patients had at least 2 positive cultures from deep biopsies and were considered as PJI. We found that the most common bacteria were *Staphylococcus epidermidis, Cutibacterium acnes,* and *Staphylococcus aureus* (Table 3).

## Discussion

We aimed to investigate the different antibiotic regimens recently used in Sweden, incidence of PJI, and which bacteria were most frequently found in PJI following primary elbow arthroplasty. We found a large variation in the use of antibiotics, that 3.6% had a PJI, and that *Staphylococcus epidermidis* was the most frequently diagnosed bacteria.

The most frequently diagnosed bacteria are the same as in other arthroplasty infections. We found *Cutibacterium acnes* as a cause of PJI, today recognized as a frequent cause of infections in the shoulder causing approximately one-fifth of the PJIs [13-15]. Other studies have identified staphylococci species and *Cutibacterium acnes* as commonly isolated bacteria in PJI of the shoulder [16,17]. Watts et al. showed in a review from 2019 that the most common causative bacteria in elbow arthroplasty are *Staphylococcus aureus* and *Staphylococcus epidermidis* but geographical variations may exist [7].

As several postoperative prosthetic shoulder infections have been described as being caused by *Cutibacterium acnes,* the addition of benzylpenicillin to cloxacillin has been used successively, but without compelling evidence [8,16,17]. It is of interest that *Cutibacterium acnes* was found in cultures in our study, because there is no information on this agent regarding elbows in the Swedish national guidelines [8].

Commonly occurring bacteria that are isolated in PJI, mainly in the knee and hip, are *Staphylococcus aureus*, streptococci, and coagulase-negative staphylococci [18].

Today, antibiotic prophylaxis is recommended for all prosthetic surgery and there are recommendations from the Swedish Infectious Diseases Association regarding antibiotic choice, doses, and length of prophylaxis [8]. However, these recommendations are not specific for elbow arthroplasty but for arthroplasties in general and based on studies of hip and knee arthroplasties [8,19].

Cloxacillin is, according to these recommendations, the first-line prophylaxis in all prosthetic surgery and is mainly directed against *Staphylococcus aureus* and methicillin-sensitive coagulase-negative staphylococci [8]. Clindamycin, which was administered at 1 hospital according to the questionnaire and in 13 patients according to the SEAR as perioperative antibiotic prophylaxis, should only be used in case of verified penicillin allergy according to a 2017 study by Robertsson et al. They demonstrated a significantly higher risk of infection related to the use of clindamycin instead of cloxacillin [20]. Another factor against clindamycin prophylaxis is a reported risk of development of resistance to clindamycin by *Cutibacterium acnes* [17,21,22].

### Strengths

Strengths of our study are that it is population-based and nationwide and, to our knowledge, the first investigation of how antibiotic prophylaxis of primary elbow replacement is conducted. We most likely included all patients treated during 2019–2021 because of comprehensive records and the combination of the 2 registers has previously shown excellent coverage.

### Limitation

Due to the low number of elbow arthroplasties and rare PJI events, no firm conclusions or recommendations concerning the best antibiotic prophylaxis can be derived from this data. It does, however, identify an area that warrants further studies, preferably prospective. Furthermore, of the 20 patients registered with a diagnosis that might indicate an infection only 11 had been tested with microbiological analysis and 2 of these were considered as superficial infections. Whether any of the other 9 had been treated with antibiotics that could potentially cure or temporarily mask a PJI is unknown but cannot be excluded and the true number of PJIs may be higher. A lower frequency of PJI in our study could be due to a shorter observation period of 2–5 years than other studies that have had slightly longer follow-ups [5,6]. Considering the relatively long interval it is, however, difficult to determine whether an infection manifested several years after the index procedure is related to the primary surgery and prophylaxis or was acquired later. There was a discrepancy between the replies to the questionnaire and the information registered in the SEAR regarding which antibiotic prophylaxis had been used during the elbow arthroplasties. The reasons for this are unknown but it could not be excluded that different people had been reporting to the respective registers, or it could be due to patient factors such as antibiotic allergy. Primary elbow arthroplasty is a relatively uncommon procedure in Sweden, with only 6 centers performing more than 4 procedures per year and centralization has occasionally been suggested. Whether inexperience had contributed to infectious complications, however, could not be investigated in the present study due to the low number of events.

Finally, a positive effect of the prophylaxis administered cannot be ascertained since an outcome of elbow arthroplasty in Sweden without any antibiotic prophylaxis is impossible to determine.

### Conclusion

We found most centers used the antibiotic cloxacillin as prophylaxis for elbow arthroplasty but with some variation. The confirmed incidence of PJI was 3.6%, which is higher than reported for other major arthroplasties. The most frequently diagnosed pathogens was *Staphylococcus epidermidis.* Our study also demonstrated that the microbiological etiology of infection was bacteria not always covered by the given prophylaxis.

*In perspective,* the lack of knowledge and consensus concerning antibiotic prophylaxis among elbow surgeons discovered in this study demonstrates the need for further research and discussion with specialists in infectious diseases to form agreement on a common strategy. Although the rate of infections may seem relatively low it is higher than reported for other major arthroplasties and any such incident constitutes a severe complication. It may be tempting to extend the antibiotic prophylaxis, but that may on the other hand increase the risk of antibiotic resistance and potential effects would need considerably larger studies to be evaluated [17,21,23].

### Supplementary data

Tables S1 and S2 are available as Supplementary data on the article page, doi: 10.2340/17453674.2025.43288

AD, JEW: data collection and analysis, manuscript preparation. BS, HBH: data analysis, manuscript review. OL: manuscript review. LEA: conceptualization, questionnaire design, data analysis, manuscript preparation.

## Notes

Handling co-editor: Jeppe Vejlgaard Rasmussen

*Acta* thanks Antti P Launonen and Anne Marie Nyholm for help with peer review of this manuscript.
